# Recent Advances in Aging-Related Diseases: Accelerated Aging, Molecular Mechanisms, Interventions, and Therapies

**DOI:** 10.14336/AD.2025.10618

**Published:** 2025-06-26

**Authors:** Elham Sadat Afraz, Seyed Alireza Hoseinikhah, Nasrollah Moradikor

**Affiliations:** ^1^Department of Oral Medicine, School of Dentistry, Semnan University of Medical Sciences, Semnan, Iran.; ^2^Emergency Medicine Physician, Victoria, Australia.; ^3^International Center for Neuroscience Research, Institute for Intelligent Research, Tbilisi, Georgia.

**Keywords:** aging, cellular senescence, mitochondrial dysfunction, anti-aging therapies

## Abstract

Aging is a multifaceted biological process influenced by cellular stress, mitochondrial dysfunction, and immune system alterations. This editorial commentary categorizes recent findings of Aging and Disease into three main areas: the acceleration of aging, prediction of age-related decline, and emerging therapeutic strategies. Research indicates that factors such as oxidative stress, chronic inflammation, and genetic predispositions contribute to premature cellular aging and the onset of age-related diseases. Recent advances in biomarkers and machine learning have improved our ability to predict biological age and associated risks, including sarcopenia and cardiovascular decline. Promising therapeutic interventions such as mitochondrial transplantation, immune system modulation, and targeted gene therapies show efficacy in decelerating aging processes and treating conditions such as Alzheimer’s disease and tissue fibrosis. A deeper understanding of these interconnected mechanisms lays the groundwork for developing personalized interventions that promote healthy aging.

## Introduction

Aging is characterized by a gradual decline in physiological functions, leading to decreased physical capacity and an increased risk of mortality. It is a major risk factor for numerous chronic diseases, such as cardiovascular disease, diabetes, neurodegenerative disorders, and cancer [1]. Multiple interconnected mechanisms contribute to the aging process and determine lifespan, such as telomere shortening, altered nutrient-sensing pathways, mitochondrial dysfunction and oxidative stress, impaired DNA repair leading to genomic instability, accumulation of misfolded proteins due to proteostasis disruption, and changes in epigenetic regulation [2]. Accurately predicting biological age is essential for the early diagnosis of age-related diseases, monitoring treatment responses, developing personalized therapeutic strategies, and deepening our understanding of the mechanisms underlying aging [3]. While several treatment approaches have been explored, recent studies have received increasing attention for their novel and promising strategies. This commentary reviews recent publications addressing the prediction and management of aging. For clarity and structure, the studies are categorized into three main themes:

### Accelerated aging

Qin et al. [4] investigated how stress hormones affect the aging process. They found that stress expedites cellular aging by activating the neuroendocrine system and increasing the release of hormones such as cortisol and adrenaline. These hormones damage mitochondria and increase reactive oxygen species (ROS), which can harm DNA and cause inflammation. These changes are linked to age-related diseases such as cardiovascular disease and diabetes. The study also mentioned some possible treatments that target pathways like nuclear factor kappa-light-chain-enhancer of activated B cells (NF-κB) and help protect mitochondria. For example, drugs like quercetin and rapamycin showed potential effects in reducing cellular damage. As a review article, the findings summarize known mechanisms but rely on previously published data, so no new experimental evidence is presented. While the overview is useful, more direct comparison between studies or evaluation of conflicting results would strengthen its impact. Also, clinical relevance is suggested (e.g., targeting sleep and mitochondrial health), but needs further validation in intervention studies.

Using AI to integrate multi-omics and imaging data may improve early diagnosis of vascular aging, but implementation in real-world settings still requires validation and cost-effective tools. The impact of stress hormones on aging and disease, as described by Qin et al., may also influence cognitive health in older adults, which Shyam et al. explored in the context of COVID-19 and lifestyle factors. Shyam et al. [5] studied the link between COVID-19 and cognitive decline in older adults with high cardiovascular risk. They used data from the PREDIMED-Plus study, which is a large trial in Spain focused on preventing heart disease through lifestyle changes. Participants were 55 to 75 years old, overweight or obese, and had metabolic syndrome. The researchers compared cognitive test results from before and after the COVID-19 pandemic. After about 50 weeks, they found no strong evidence that COVID-19 caused decline in most cognitive areas. In fact, there was a small improvement in overall cognitive function, especially among those who followed healthy lifestyle habits like a Mediterranean diet and regular exercise. These results suggest that any short-term effects of COVID-19 on thinking skills may be temporary and could improve with a healthy lifestyle. However, as this was not a longitudinal or interventional study, we can't say poor sleep causes myelin loss—it may be the other way around. Also, since participants were all from one Japanese cohort, results may not generalize to other ethnic or cultural groups. Still, the research suggests that improving sleep may help protect brain structure and function in aging, which is an important area for future interventions.

Zhang and others [6] reviewed how aging of blood vessels is linked to diseases like atherosclerosis and Alzheimer’s. They focused on important biological pathways such as mechanistic target of rapamycin (mTOR), AMP-activated protein kinase (AMPK), nuclear factor kappa-light-chain-enhancer of activated B cells (NF-κB), sirtuin (SIRT), and Klotho, along with biomarkers like β-galactosidase and exosomal miRNAs that are involved in vascular aging. The review showed that reducing oxidative stress and improving mitochondrial and telomere function could help slow down blood vessel aging. The authors also emphasized that new tools like artificial intelligence and machine learning may improve how we diagnose and treat vascular diseases in older adults. In addition, the paper mentioned that sleep quality may also play a role in how fast or slow blood vessels age.

Building on the role of vascular aging in cognitive decline, recent studies have also highlighted how factors like sleep quality affect brain structure and function in older adults. A recent cross-sectional study on older Japanese adults (aged 65-82) showed that poor sleep quality is linked to lower myelin levels in the brain, especially in fronto-temporo-parietal and limbic regions [7]. This reduction in myelin was also associated with weaker cognitive function and higher levels of depression. Participants were grouped based on their sleep quality using the Pittsburgh Sleep Quality Index (PSQI), and advanced MRI scans were used to measure brain myelin. By matching both groups for age, education, and health factors, the researchers aimed to minimize confounding effects. These findings highlight the importance of good sleep for maintaining brain health in older age. However, as the study was cross-sectional and limited to a specific Japanese cohort, more research is needed in diverse populations and using longitudinal designs to confirm whether improving sleep quality can causally improve brain structure and function. Understanding these mechanisms could guide future interventions to maintain cognitive health during aging, possibly by targeting sleep and related metabolic or inflammatory pathways. In addition to brain health, maintaining mitochondrial function is also crucial for other sensory systems affected by aging, such as hearing. It has been reported that the gene Cisd2 plays an important role in protecting mitochondrial function and maintaining cochlear health [8]. In this study, neuron- and cochlea-specific Cisd2 knockout mice were used, and their hearing was tested with auditory brainstem response (ABR) and distortion product otoacoustic emissions (DPOAE) to evaluate auditory function. Loss of Cisd2 leads to worsening hearing loss by disrupting potassium balance and synaptic activity in the ear. Although the results support the role of Cisd2 in hearing, the study only used male mice, which may limit the generalizability of the findings. Furthermore, the effects of increasing Cisd2 expression were not explored. The human sample size was small, and more evidence is needed to confirm the relevance of Cisd2 in human hearing loss. This research suggests that targeting Cisd2 could be a promising strategy for preventing or treating age-related hearing loss by protecting mitochondrial and synaptic functions in the cochlea. Both mitochondrial function and cellular aging processes, like senescence, play key roles in the decline of sensory and brain health during aging.

Cellular senescence causes to irreversible cell cycle arrest and alters cell function through the secretion of the senescence-associated secretory phenotype (SASP), as reported by Feng et al. [9]. In the brain, senescent neurons, glial cells, and immune cells contribute to changes in immune responses and overall brain health, influencing development, aging, and neurodegenerative diseases. Although the review mentions important mechanisms and implications of senescence, it lacks original experimental data and relies on previously published studies, limiting direct evidence for some claims. The review does not fully address potential variability between different brain regions or cell types. Despite these limitations, understanding cellular senescence offers promising avenues for developing novel therapies targeting age-related and neurodegenerative central nervous system disorders. Cellular aging and mitochondrial dysfunction are closely linked processes that together influence brain and liver health during aging, especially under stress factors like parental alcohol exposure.

Basel and others [10] studied tissues at 42 weeks old, using scans, different staining methods, gene expression tests, and protein analysis to understand these effects. They also looked at differences between males and females, and between mothers’ and fathers’ alcohol use. They showed that when parents consume alcohol, their offspring experience lasting mitochondrial problems and early signs of aging in the brain and liver. Mice exposed to alcohol from both parents had the worst liver damage. Although the study gives strong evidence that alcohol speeds up aging by affecting mitochondria and NAD^+^ metabolism, it does not explore how these changes affect behavior or long-term health. This work showed possible ways to treat or prevent alcohol-related aging problems by targeting mitochondria and metabolism. Regarding the role of mitochondria in aging, recent research has also focused on mitochondria-associated membranes (MAMs), which play a crucial role in regulating cellular processes linked to aging and disease. Wang and others [11] reviewed MAMs as key regulators of calcium signaling, lipid metabolism, and autophagy. They showed that changes in MAM structure and function are linked to aging and related diseases by disrupting mitochondrial activity and cell survival. However, the review is mostly based on indirect evidence, and direct experimental data on how MAMs control aging processes remain limited. More targeted studies are needed to clarify these mechanisms. Despite this, their work suggests that modulating MAM dynamics may offer promising therapeutic strategies for age-related conditions. Based on mitochondrial functions, aging-related conditions also involve complex systemic changes, such as those seen in diseases like myotonic dystrophy type 1, which share features with frailty.

In addition, Garmendia et al. [12] reported that myotonic dystrophy type 1 (DM1) shares several characteristics with frailty, fulfilling at least four of Fried’s frailty criteria. Their longitudinal analysis demonstrates that these frailty features worsen over time, supporting the concept of accelerated aging in DM1 patients. Additionally, psychological and social factors were identified as important contributors to frailty risk. However, as a narrative review, the study is based on existing data and lacks new experimental evidence, which limits the strength of causal conclusions. Nonetheless, their findings emphasize the importance of monitoring frailty in DM1 to enable earlier interventions and potentially reduce disease burden and adverse outcomes. Building on the understanding of frailty and its impact on aging-related diseases, recent research has also focused on cellular senescence as a key driver of aging and a target for novel immunotherapies. Wang et al. [13] highlighted cellular senescence as a central feature of aging that contributes to age-related diseases, and noted that removing senescent cells can delay aging processes. They reviewed emerging immunotherapies, such as immune checkpoint inhibitors and engineered immune cells like CAR-T, CAR-NK, and CAR-M, which show potential for selectively targeting senescent cells. However, challenges such as treatment toxicity, high costs, and the lack of specific senescence biomarkers limit current applications. The authors suggest that dual-target CAR approaches and combination therapies may improve both specificity and effectiveness. Although promising, these immuno-therapies require further development and validation before they can be broadly applied to extend healthy lifespan. Building on the idea of senescent cells, Xiao et al. [14] looked at how old macrophage cells cause inflammation and damage in aging. Xiao et al. [14] reviewed the role of macrophages in promoting chronic inflammation during aging, a phenomenon often termed “inflammaging.” They explained that aging induces macrophage senescence, leading to the secretion of pro-inflammatory factors collectively known as the senescence-associated secretory phenotype (SASP). This contributes to increased inflammation and tissue damage in aged organs. The studies show that aging is accelerated by stress, mitochondrial damage, inflammation, and environmental exposures such as alcohol. Hormones like cortisol and adrenaline increase oxidative stress, leading to DNA damage and cellular senescence. Diseases such as COVID-19 and DM1 are associated with aging-like symptoms, including frailty and cognitive decline. Factors like sleep quality, hearing loss, and vascular changes also contribute to accelerated aging, primarily by impacting brain and mitochondrial health. Senescent cells and immune dysfunction further exacerbate inflammation (inflammaging) and tissue damage.

### Prediction

Welch et al. [15] studied factors predicting acute sarcopenia in patients aged 70 and older with infections. The study was conducted at a single hospital in the UK, including patients for elective or emergency surgery and those admitted with infections. Muscle mass and strength were measured with the help of handgrip strength, ultrasound, and bioelectrical impedance analysis (BIA) at different time points after admission and/or surgery. They found that conditions such as chronic obstructive pulmonary disease (COPD), steroid use, delirium, and higher levels of interleukin-1 beta (IL-1β) and interleukin-7 (IL-7) increased the risk of rapid muscle loss and reduced muscle quality within a week. However, no single reliable biomarker was identified to predict acute sarcopenia. It highlights the need for more research. Although the study highlights important risk factors, it is limited by its single-center design and lack of a clear predictive biomarker. Larger studies are needed to confirm these findings.

Identifying patients at risk could allow earlier interventions to prevent muscle loss in older adults after illness or surgery. Early identification of physical decline in older adults, such as acute sarcopenia, complements advances in cardiovascular aging assessment, together improving overall aging management. A study developed EchoAGE, a neural network model estimating heart biological age using echocardiographic data from over 5,000 Caucasian patients [16]. Echocardiography data were collected using Philips and GE ultrasound devices, and the model was trained on data from patients without age-related diseases, then tested on those with various conditions. The model showed high accuracy (MAE ≈ 3.5 years, R^2^ ≈ 0.88) by analyzing heart structure and function parameters like the E/A ratio, wall thickness, and cardiac output. EchoAGE can help detect early aging-related heart changes, aiding risk prediction for age-associated diseases. However, the model needs validation in diverse populations and may require high-quality imaging. It limits its use in some settings. Despite these limitations, EchoAGE suggests a promising method for early cardiovascular aging diagnosis and prevention.

### Treatment

Zhao et al. [17] reviewed engineered mitochondrial transplantation as a potential anti-aging therapy. They emphasized that mitochondrial dysfunction plays a central role in aging and suggested the transfer of healthy mitochondria into damaged cells as a novel therapeutic strategy. This method may restore mitochondrial function and suggests benefits for age-related diseases such as neurodegeneration and cardiovascular disorders. The review covered mitochondrial structure and properties, dysfunction mechanisms, intercellular mitochondrial transfer, and its potential application across different systems, such as the skin. While pre-clinical studies show promise, clinical research is still in its early stages. This article highlights an innovative and biologically targeted method for combatting aging, which is consistent with the root causes of cellular decline. However, limitations remain, including technical challenges in delivery, immune response risks, and lack of standardized protocols. If optimized, mitochondrial transplantation could represent a significant advance in regenerative and anti-aging medicine. With regards to mitochondrial-focused therapies, other molecular targets such as cathepsin enzymes also show promise for treating age-related neurodegenerative diseases.

Liu et al. [18] reviewed the potential of cathepsin enzymes as therapeutic targets in Alzheimer’s disease (AD). They discussed how cathepsins regulate protein degradation, neuroimmune balance, and inflammation. Dysregulation of these enzymes may contribute to beta-amyloid and tau accumulation, which are central to AD pathology. The authors suggested that modulating specific cathepsins could suggest new strategies for early diagnosis and treatment. This review also showed related topics such as inflammation signaling, mitochondrial dysfunction, and danger-associated molecular patterns (mtDAMPs) in cardiovascular diseases (CVDs), highlighting overlapping mechanisms between CVD and AD. The study presents a promising direction by linking lysosomal biology to neurodegeneration. However, translating these findings into treatments requires more in vivo validation and specific cathepsin modulators with minimal off-target effectsCathepsin-targeted therapy may help both cognitive and systemic aging disorders. Complementing enzyme-targeted methods, mitochondrial transfer therapies suggest another promising way to restore cellular function in neurodegenerative diseases. In a review article, Zhou et al. [19] reviewed the therapeutic potential of mitochondrial transfer within the neurovascular unit (NVU) for central nervous system (CNS) diseases. They showed how mitochondria-either transferred naturally between brain cells or through transplantation-can help restore cellular energy and function, especially in disorders with mitochondrial dysfunction. Astrocytes, neurons, microglia, endothelial cells, and pericytes all participate in mitochondrial exchange by mechanisms such as tunneling nanotubes, extracellular vesicles, and gap junctions. The authors suggested that combining internal and targeted mitochondrial delivery may improve precision in treating neurodegenerative diseases. This review provides a comprehensive understanding of mitochondrial dynamics and transfer in brain health. In despite mechanistic insights are promising, translating them into clinical practice faces challenges in delivery methods, targeting accuracy, and immune response. Mitochondrial transfer presnts an innovative and cell-specific strategy with high potential in CNS repair. In addition to mitochondrial transfer’s role in restoring cell function in neurodegenerative diseases, proteins such as SIRT2 that depend on NAD^+^ are also important in aging-related tissue damage and fibrosis. A recent review by Huang et al. [20] reviewed the role of SIRT2, a NAD^+^-dependent deacetylase, in age-related fibrosis, focusing on the liver, kidneys, and heart. SIRT2 affects several pathways involved in inflammation, tissue remodeling, and fibrosis progression. It interacts with key signaling molecules (e.g., AKT, p53, NLRP3), affecting diseases such as HBV-related liver fibrosis, renal injury, and cardiac hypertrophy. The review also discussed SIRT2 inhibitors as potential therapies. While SIRT2 shows therapeutic promise, its effects vary between organs. It suggests the need for organ-specific research. Better understanding could help design targeted anti-fibrotic strategies in aging. In addition to aging-related fibrosis in organs such as the liver and heart, cellular dysfunction also plays an essential role in chronic inflammatory diseases such as rheumatoid arthritis, where specific cell types contribute to disease progression.

In a review study, Zhang et al. [21] highlighted that synovial fibroblasts (SFs) play an active role in the development of rheumatoid arthritis (RA). They reported specific SF subsets that contribute to persistent inflammation and immune system activation. SFs were shown to affect both innate and adaptive immune responses, including T and B cell interactions. The study highlighted that changing the ratio of these SF subtypes could help reduce RA severity. While targeting SFs is a promising strategy for RA therapy, challenges remain in achieving specificity without affecting normal tissue function. Further research may cause safer, fibroblast-directed treatments for chronic joint inflammation. In addition to the role of fibroblasts in chronic inflammation, targeting specific molecular pathways in brain regions also shows promise for treating age-related diseases such as Alzheimer’s. Iqbal and others [22] in an animal study, reported that silencing the TGFβR II gene specifically in the retrotrapezoid nucleus (RTN) of AD model mice improved memory and respiratory function. Using lentiviral vectors targeting astrocytes, the intervention reduced GFAP expression in the RTN and decreased amyloid-β burden in both the cortex and hippocampus. Behavioral tests showed increased cognition and fewer apneas following treatment. While the results are promising, the targeted delivery to the brainstem limits immediate clinical translation. Nonetheless, the study highlights RTN inflammation as a potential therapeutic target for addressing both cognitive and respiratory symptoms in AD. Chen et al. [23] reviewed the mechanisms of immune checkpoint inhibitor (ICI) therapy-induced arthritis, highlighting the central role of T cells infiltrating joints and releasing inflammatory mediators. They reported that ICI arthritis varies clinically and immunologically from autoimmune arthritis. It shows an imbalanced T cell population with increased CD8^+^ and decreased regulatory T cells, chnaged gene expression, and heightened IFN sensitivity in joint T cells. The study proposed targeted strategies, including selective ICI regimens, anti-interleukin therapy, JAK inhibitors, and modulation of T cell differentiation or migration, to decrease joint inflammation while preserving anti-tumor immunity. These findings suggest the way for safer cancer immunotherapy with minimized autoimmune side effects.

While Chen et al. focused on immune mechanisms and inflammation in therapy-induced arthritis, the following review expands on molecular regulators, specifically antisense long non-coding RNAs, that affect inflammation and pathology in AD [24]. AS-lncRNAs regulate critical AD-related genes implicated in amyloid-beta (Aβ) aggregation and tau protein pathology. The study showed their involvement in neuroinflammation, synaptic plasticity, neuronal apoptosis, and oxidative stress, all contributing to AD progression. Additionally, AS-lncRNAs kept promise as diagnostic biomarkers and therapeutic targets, though challenges remain in translating these findings into clinical applications. The review also showed future research directions to better understand AS-lncRNA functions and develop novel AD treatments. The review clearly shows the potential role of AS-lncRNAs in AD, but direct evidence of their exact functions in the human brain is still limited. More clinical studies are needed to understand their mechanisms and to explore their use as reliable diagnostic markers or treatment targets. BACE1-AS and MAPT-AS1 are promising biomarkers or drug targets. It has also been reported that nerve growth factor (NGF) has significant potential in treating conditions like stroke and trauma by aiding nerve repair [25]. However, its use in neurodegenerative diseases is still limited due to delivery challenges and complex pathology. New strategies, such as gene therapy and neuromodulation, may improve NGF-based treatments in the future. Faraji and Metz [26] reported brain-derived neurotrophic factor (BDNF) helps brain cells survive, and flexibility during aging. BDNF may help slow brain aging and support thinking and mental health. Since BDNF works differently in each brain area, using it in specific regions may work better than affecting the whole brain. Long-term studies are needed to find the best ways to use BDNF for brain health in older people. Stroke can damage brain cells by cutting off blood flow. GeNL imaging shows low-oxygen areas in the brain after stroke that affects recovery. With regards to the importance of supporting brain health during aging, Zhou et al. focused on protecting brain cells after stroke by monitoring oxygen levels, which is crucial for recovery and preventing further damage.

Zhou et al. [27] proposed that controlling oxygen levels in the brain after a stroke could help protect brain cells and improve recovery. They introduced a new tool called GeNL that could detect small areas of low oxygen in the brain more accurately than older methods. This tool helps researchers understand how oxygen levels change during and after a stroke, and how these changes affect brain function. The study also discussed the limitations of past techniques and highlighted the requirement for better tools to study brain oxygen. Although GeNL shows promise, more research is needed to confirm its use in clinical settings. This approach could cause more personalized and effective treatments for stroke patients. Following advances in brain oxygen monitoring after stroke, recent research has also focused on molecular modifications such as lactylation, which play important roles in inflammation and brain function. It suggests new insights into disease mechanisms and potential therapies. Lactylation is a newly discovered post-translational modification that depends on tissue lactate levels and helps control how proteins work and how genes are expressed. Studies have shown that lactylation can affect not only histones but also many non-histone proteins. This influences key processes such as inflammation, immune response, brain function, and cancer development [28]. The reviewed study explored this by analyzing global patterns of lysine lactylation and linking it to many disease processes, especially in inflammation, tumors, cardiovascular health, and the brain. It plays important roles in several diseases, such as cancer, heart problems, and mental health disorders.

Understanding how lactylation works may help researchers find new ways to treat a wide range of diseases. Li et al. [29] reviewed new disease-modifying therapies for Alzheimer’s disease that aim to clear amyloid using both active and passive immunization. While animal studies have shown positive results, active immunization in humans caused side effects, which limits its clinical use. Passive approaches, such as the FDA-approved antibodies aducanumab and lecanemab, show some promise but offer only modest benefits. The review also discussed innovative treatments involving T cells and CAR-based strategies, which may provide more targeted effects in the future. However, these newer methods still face safety concerns and need further testing. Although the focus on the immune system opens up exciting treatment options, more studies are needed to improve effectiveness and reduce risks before these therapies can be widely used in patients. Based on advances in AS treatments, new healthcare models like longevity clinics are emerging to focus on early prevention and personalized treatment, aiming to improve healthspan and delay age-related diseases. Mironov et al. [30] studied longevity clinics and reported them as a new healthcare model focused on preventing diseases before symptoms appear. These clinics use aging clocks and biomarkers to help people live healthier for longer. The model is based on an Analytical Center that supports data-driven research, personal treatment plans, and ongoing evaluation of medical tools. By working with scientific longevity centers, these clinics aim to develop precise therapies, support clinical trials, and provide affordable, high-quality care. This method could play an important role in improving healthcare for aging populations. While longevity clinics focus on early prevention and personalized care to extend healthy lifespan, understanding cellular aging processes such as reduced mitophagy in endothelial cells is crucial for developing targeted therapies to maintain cardiovascular health during aging. Han et al. [31] reviewed how aging affects endothelial cells, which are essential for maintaining blood vessel health. They found that aging reduces mitophagy, the process that clears damaged mitochondria, causing their buildup in cells. This leads to poorer blood vessel function and contributes to cardiovascular diseases. The study highlighted that boosting mitophagy in endothelial cells may help prevent or treat age-related heart problems. However, most evidence comes from preclinical models, and more human studies are required to confirm its effectiveness. Understanding these mechanisms is practically important for developing targeted treatments to support healthy aging of the cardiovascular system.

In summary, new therapies aim to reverse or slow aging by targeting key biological systems. Mitochondrial transplantation and internal mitochondrial transfer show promise for treating age-related diseases such as Alzheimer’s and heart disease. Other methods focus on modulating inflammation, immune responses, or genetic regulators like SIRT2, BDNF, and AS-lncRNAs. Advances in immunotherapy, fibroblast targeting, and NGF delivery suggest potential treatments for arthritis, stroke, and neurodegeneration. Longevity clinics and aging clocks support personalized care and early prevention. Increasing mitochondrial quality, immune balance, and cellular repair mechanisms may provide effective anti-aging strategies.

### Conclusion

Aging is affected by multiple biological processes, such as cellular senescence, mitochondrial dysfunction, and chronic inflammation, all of which can accelerate disease onset and tissue decline. Identifying early predictors, such as inflammatory markers, heart aging models, and frailty indicators-can help detect aging-related risks before symptoms appear. Emerging treatments, such as mitochondrial therapies, immunotherapy, and gene-targeting approaches, suggest hope for reversing or slowing age-related damage. Combining early detection tools with targeted therapies supports a personalized approach to aging care. Continued research is essential to improve safety, accuracy, and effectiveness. Together, these strategies aim not only to extend lifespan but also to increase quality of life in aging populations.
